# Ultrastructural localization of the likely mechanoelectrical transduction channel protein, transmembrane-like channel 1 (TMC1) during development of cochlear hair cells

**DOI:** 10.1038/s41598-018-37563-x

**Published:** 2019-02-04

**Authors:** Shanthini Mahendrasingam, David N. Furness

**Affiliations:** School of Life Sciences, Keele University, Keele, Staffordshire ST5 5BG UK

## Abstract

Transmembrane channel like protein 1 (TMC1) is likely to be a pore-forming subunit of the transduction channel of cochlear hair cells that is mechanically gated by tension on tip links in the stereocilia bundle. To localise TMC1 precisely, we labelled mice cochleae of different ages using custom-made polyclonal antibodies to TMC1 for light and transmission electron microscopy (TEM). Immunofluorescence revealed stereocilia labelling at P9 but not at P3 in apical hair cells. Immunogold labelling for TEM confirmed that labelling was absent at P3, and showed weak labelling at P6 with no stereocilia tip labelling, increasing at P9, with specific tip labelling on shorter stereocilia and some throughout the bundle. At P12 and P21, labelling was refined mostly to stereocilia tips. Quantification showed that labelling overall reached maximum by P12, labelling per tip was relatively constant from P9 to P21, but percent tips labelled was reduced from 16% to 8%. *Tmc1*^−/−^ showed no labelling. Thus TMC1 occurs at the lower end of the tip link, supporting its presence in the MET complex and likely the channel. Tip localisation from P9 onwards coincides with lipoma HMGIC fusion partner-like 5 (LHFPL5), a protein that may be involved in acquiring/maintaining TMC1 localisation.

## Introduction

Hair cells of the cochlea have an apical hair bundle containing a highly sensitive mechanoelectrical transduction (MET) apparatus that enables them to detect mechanical stimulation caused by sound. The bundle consists of rows of actin-filled stereocilia ranked in a staircase fashion, with the height of each successive row increasing from short to tall, linked together by multiple extracellular filaments. A single microtubular kinocilium occurs to one side of the bundle during development but is not present in adults. MET is associated with the tip link, a filament that connects each shorter stereocilium to the side of the taller stereocilium in the row behind (see review^1^). A complex located at the lower end of the tip link contains MET channels and accessory proteins. The channels are gated by changes in tip-link tension when the bundle is deflected, causing the hair cell to depolarize and hyperpolarize with the cycles of acoustic stimulation (see review^2^). The two kinds of cochlear hair cell, inner (IHC) and outer (OHC) are fundamentally similar in how they transduce, although there are physiological and structural differences between them^1,2^.

There are ongoing efforts to understand the composition and organisation of the MET apparatus. The tip link contains two cadherin related proteins, protocadherin 15 (PCDH15) at its lower end and cadherin 23 at its upper end (CDH23)^3^. Associated with the lower end of the tip link, i.e. specifically with PCDH15, are proteins such as lipoma HMGIC fusion partner-like 5 (LHFPL5^4,5^), transmembrane protein of the inner ear (TMIE^6^), two likely channel components, transmembrane-like channel 1 (TMC1) and TMC2^7–9^, and calcium sensitive integrin-binding protein 2 (CIB2^10^), amongst others. Mutations in these proteins are associated with deafness in mice models and humans, for example mutations in mice with hearing loss such as *deafness (dn)*^11^ and *Beethoven (Bth)* are known to affect the *Tmc1* gene^12^. Mutations in human *TMC1* cause *DFNB7/11*^13,14^ and *DFNA36*^13,15^, common forms of genetic deafness in some populations; Usher Syndrome type I reflects mutations in tip link proteins^16,17^ that cause tip-link loss in mouse models^18^.

Evidence that TMC proteins form the channels comes from double knockout of TMC1 and TMC2. This abolishes conventional MET currents completely, whilst in early postnatal cochleae knocking out TMC1 alone does not, although MET is lost later in development^7^. This has been taken to imply TMC2 can form MET channels in early stages and can substitute for TMC1, a possibility confirmed by the fact that TMC1/TMC2 double knockout can be, to some degree, rescued by exogenous expression of either TMC1 or TMC2^7^. TMC1 and TMC2 appear to be developmentally segregated so that whilst both proteins occur in stereocilia at P3, TMC2 declines whilst TMC1 remains in adults^19^. Biochemical evidence suggests TMC1 assembles as a dimer, and cysteine mutagenesis followed by expression of the mutant TMC1 in hair cells of *Tmc1/2*-null mice indicate it forms the pore region^9^. In addition, gradients in the MET channel conductance occur at P5 along the tonotopic axis (representing the gradation in frequency detected) of the cochlear spiral that have been attributed to changes in the number of TMC1 molecules at stereocilia tips, based on experiments using mCherry-TMC1 expression^20^.

Data from Zhao *et al*.^6^ suggested associations can occur between LHFPL5, TMIE and PCDH15. TMC proteins interact with PCDH15^21^ and TMC1 knockouts affect the distribution of LHFPL5^22^. We have shown that LHFPL5 is distributed throughout the bundles, including all stereocilia tips, in early development but becomes refined in mice by postnatal day 12 (P12)^5^, coincident with the onset of hearing. The same type of data do not yet exist for TMC1.

To understand better the role of TMC1, it is important to establish its precise location and developmental acquisition in the hair bundle, in relation to other salient proteins and structures, such as the tip link. Localisation studies using confocal microscopy suggest that it is present in the tip region of the short stereocilia^19^. However, standard confocal microscopy lacks the resolution to observe fine structural features of the hair bundle and thus obtain precise localization. In the present study, we have employed pre-embedding immunogold electron microscopy to evaluate the distribution of TMC1 at the ultrastructural level during development.

## Results

### Antibody generation and validation

Antibodies to TMC1 were generated to the peptide CDEETRKAREKERRRRLRRGA (aa 53–72 from the mouse TMC1 protein) in two separate rabbits (AB0144 and AB0145). The serum was affinity purified against the peptide and used for immunofluorescence in the first instance, for validation against wild-type and knockout mice. Preliminary immunofluorescence showed that the most consistent of these antibodies was AB0144. This antibody was therefore used to label samples for all subsequent experiments.

**Figure 1:**
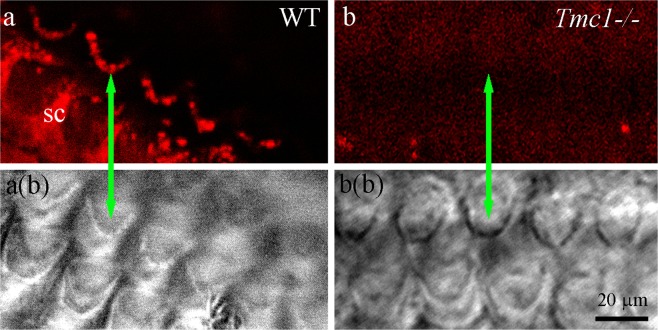
Immunofluorescence labelling and confocal imaging of wild type (WT) and TMC1 knockout (*Tmc1*^−/−^) OHC bundles from apical cochlear region at P12. (**a**) Stereocilia are labelled along with the apices of supporting cells (SC). (**b**) Neither cell type is labelled in the knockout control. Phalloidin was not used as a counterstain, but bundle localization is evident from the equivalent bright field images a(b) and b(b). Green arrows identify the same bundle in each pair of images.

Initial confocal imaging of immunofluorescent labelled P12 wild-type samples showed hair bundle labelling and some supporting cell labelling. Phalloidin-FITC, a fluorescent actin stain often used to label stereocilia for morphological identification, was omitted from these samples because the strength of the signal sometimes caused bleed-through from the green phalloidin-FITC channel to the red channel on the confocal system. To check for unambiguous labelling therefore, labelling only with primary antibody was performed (Fig. 1a). Transmitted light observations at the same level of focus, although not displaying confocality, were sufficient to show the presence of hair bundles across the plane of focus of the confocal images. In P12 knockout samples, both hair bundle and supporting cell labelling were eliminated, again confirmed with transmitted light imaging at the plane of focus of the bundle (Fig. 1b).

The positive labelling was sensitive to fixation: the most consistent labelling was obtained with a short 4% paraformaldehyde fixation step of 20 min. In addition the labelling was sensitive to the age of the antibody from when it was taken out of −20 °C storage: the shorter the period between defrosting and use, the more consistent the labelling.

With the knockout validation, we then performed immunofluorescence at P3 and P9 to bracket the likely developmental acquisition period of the TMC1. Phalloidin-FITC was included in these experiments as imaging of smaller, more immature bundles, especially at P3, was difficult using transmitted light detection.

**Figure 2:**
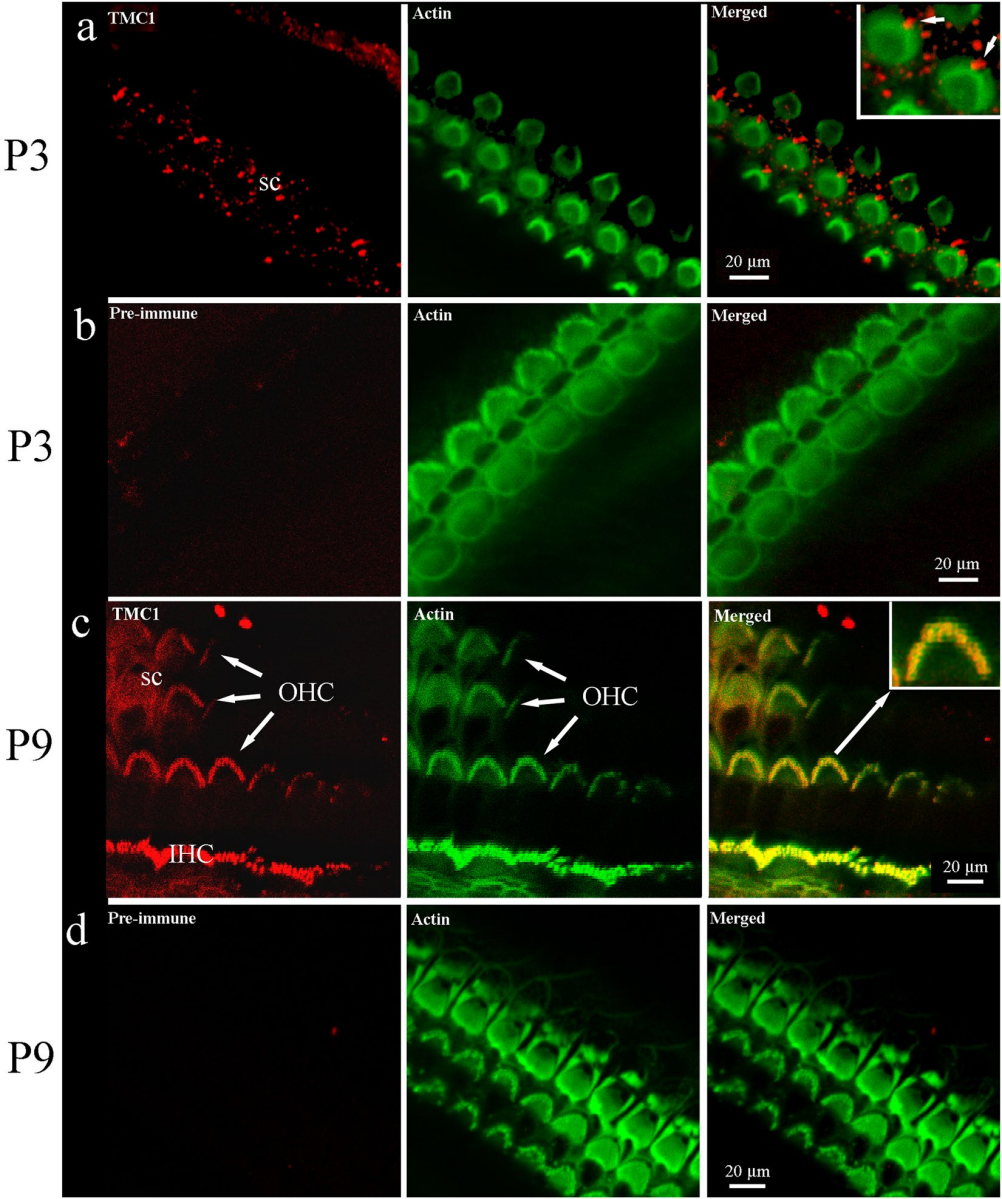
Immunofluorescent confocal imaging of organ of Corti from the apical cochlear region at P3 and P9. (**a**) TMC1 is not detected on stereocilia at P3, although the kinocilium appears labelled (arrow in merged image, inset), and speckled labelling is noted on supporting cells. Phalloidin confirms the presence of hair bundles at the same focal plane. (**b**) Pre-immune serum did not generate any labelling at P3. (**c**) TMC1 labelling was detected in IHC and OHC bundles at P9; colocalisation with stereocilia is observed as a yellow colour in the merged image. Weak fluorescence was detected on the hair-cell apical surface. The inset shows an enlargement that reveals red and green signal in the indicated (arrow) bundle can also be distinguished. (**d**) Pre-immune serum did not produce labelling. In all red channel images, the green channel was subtracted using the confocal software to remove bleed through from the phalloidin staining.

At P3, IHC and OHC hair bundle labelling was absent (Fig. 2a). Weak bleed through of the phalloidin signal was removed by subtraction of the green channel, leaving residual speckled red labelling in the supporting cells, but no stereocilia labelling. However, bright red spots were detected, often at the vertices of the hair bundles, coincident with the location of the kinocilium, an accessory cilium with a microtubular axoneme that is present during cochlear hair bundle development but lost in adults (Fig. 2a). Supporting cell labelling was also evident at P3. Pre-immune serum was used as a control, by replacing the primary antibody, and generated no labelling in the P3 samples (Fig. 2b).

At P9 strong labelling of all three OHC and one IHC rows, and weak hair-cell and supporting cell surface labelling, was detected and confirmed by subtraction of the green channel (Fig. 2c). For the larger IHC stereocilia, labelling appeared in the middle region of the bundle, although specific localization to the tips was difficult to confirm. No labelling was detected on tall stereocilia tips. Pre-immune serum was used as a control, by replacing the primary antibody, and generated no labelling in the P9 samples (Fig. 2d).

### Pre-embedding immunogold labelling

**Figure 3:**
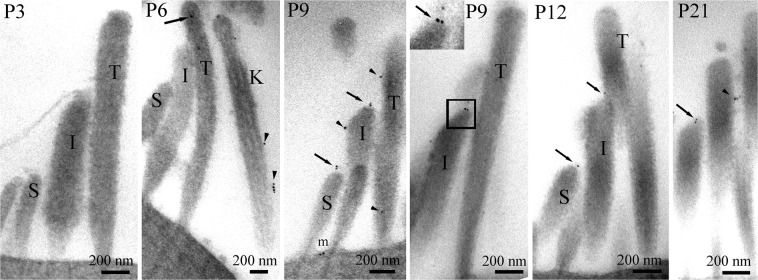
Immunogold labelling of OHC stereocilia bundles from apical cochlear regions. Gold particles could not be detected at P3 but were present in small quantities, apparently randomly in the bundle (arrows) and on the kinocilium (arrowheads) at P6. By P9, gold particles were observed on the tips of stereocilia in a position coinciding with the lower end of the tip link (arrows, inset) and occasionally on the shafts (arrowheads). At P12 and P21 tip labelling continued to be present whilst shaft labelling was sparse (arrowheads). Some apical membrane labelling was detected at all ages, as seen for example at P9 (m).

The P3–P9 immunofluorescence comparison showed that, when using the custom made antibody AB144 to detect it, TMC1 is acquired in the hair bundle sometime after P3 and before P9 in the apical turn of the mouse cochlea. We therefore performed pre-embedding immunogold labelling at P3, P6, P9, P12 and P21. The advantage of the pre-embedding method is that it is high resolution and semi-quantitative, i.e. we can analyse distributions within bundles in defined stereociliary rows, identify tips where tip links are likely to emanate from, and compare numbers of particles per bundle over time to indicate how much the relative expression of the protein changes. We focused on OHC bundles from apical cochlear regions as it is easier to find these undisturbed compared with IHCs, and to assign stereocilia to different row heights. In development we can thus identify short, intermediate and tall rows clearly (Fig. 3).

At P3 no bundle labelling was observed whilst at P6 a few gold particles were present but not localized in any specific region of the stereocilia (Fig. 3). At P9, P12 and P21, some sparse labelling was expressed on stereocilia shafts, but a proportion of short and intermediate stereocilia tips were also labelled. Rarely were tall stereocilia tips labelled. Labelling was also consistently detected on the membrane at all ages, as illustrated for P9 (Fig. 3).

**Figure 4:**
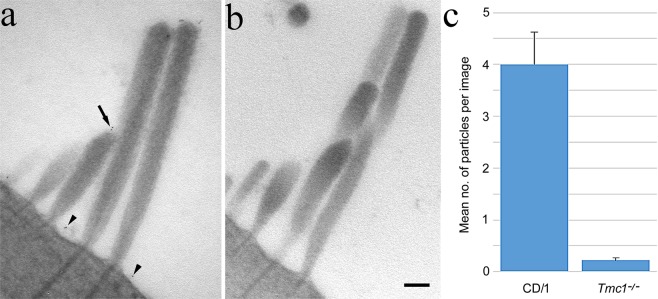
Simultaneous immunogold labelling of (**a**) wild type and (**b**) *Tmc1*^−/−^ OHC bundles from apical cochlear regions, and (**c**) gold particle counts (mean ± SEM, n = 3) from both at P10. Virtually no labelling was detected on *Tmc1*^−/−^ bundles whereas labelling on wild type was present as before at shorter stereocilia tips (arrow) and on the apical membrane (arrowheads). The mean gold particles per bundle was significantly different between the two [Wilcoxon (Mann-Whitney) test, *p* < 0.05].

Pre-embedding immunogold labelling was also performed simultaneously on 3 WT and 3 *Tmc1*^−/−^ animals at P10. In *Tmc1*^−/−^, virtually no bundle labelling was observed; quantitatively there was a significant difference in bundle labelling in about 50 images per sample between the two (Fig. 4).

**Figure 5:**
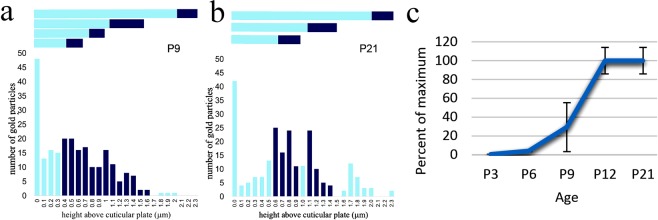
(**a**,**b**) Graphs showing the distribution of particles from the apical membrane to the tips of the tallest stereocilia in OHC bundles from the cochlear apex at P9 and P21. The lengths of each row as determined across ~50 images is shown horizontally above the histogram: dark blue bars represent the tip regions. (**a**) At P9, particles were found relatively evenly spread through the shorter rows of stereocilia. (**b**) By P21, labelling was refined to coincide mainly with the tips of short and intermediate stereocilia. (**c**) Graph showing n/n(max)% particles in approx. 50 images of OHC bundles at different ages. The n/n(max)% is almost 0 at P3 but rises through P9 to reach 100% at P12 which is maintained at P21. The high variance at P9 is indicative of one higher and two lower values.

To assess the changes in particle distribution in bundles between P9 (pre-hearing) and P21 (post-hearing onset, weaned animals), the number of particles was scored against height in bins above the apical membrane across approx. 50 images and plotted as histograms. This showed that at P9, particles were distributed throughout the bundle, with a high signal on the hair-cell apical membrane and a small enrichment towards the tip regions of the rows of shorter and intermediate stereocilia (Fig. 5a). By P21, a small amount of residual labelling was observed in the lower shafts, but most particles were either on the apical membrane or in the vicinity of short and intermediate stereocilia tips (Fig. 5b).

**Figure 6:**
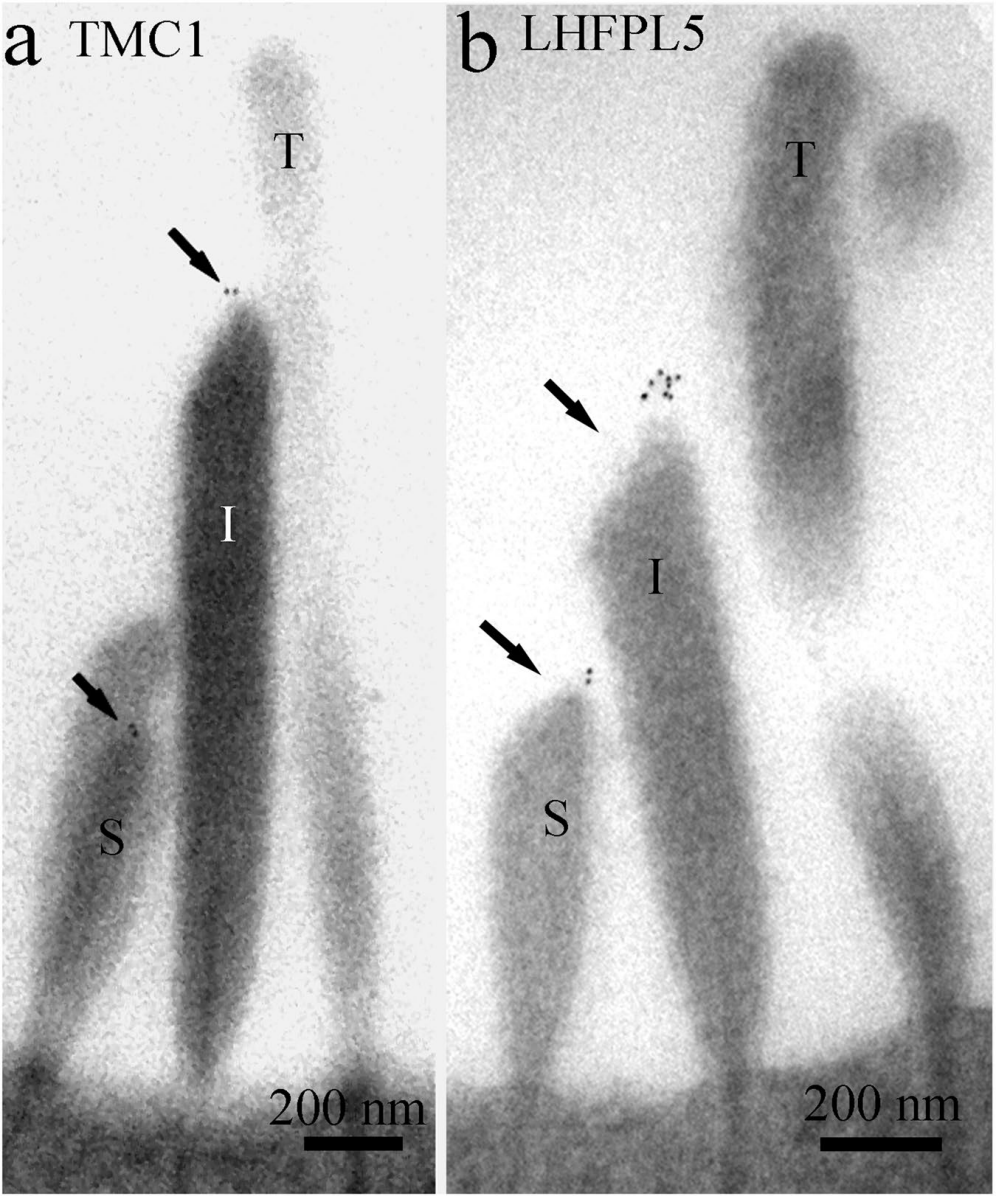
Comparison of TMC1 (**a**) and LHFPL5 (**b**) labelling in OHC bundles from apical cochlear regions at P12. The labelling patterns are similar. Note that in **a** one short stereocilium is superimposed on a slightly taller one; the particles seen are at the tips of the shorter of these two (arrows).

A semi-quantitative analysis of developmental acquisition was performed by counting numbers of particles per bundle, the proportion of tips labelled, and the number of particles per labelled tip in approx. 50 images from OHC bundles from P3 to P21. It was clear from this that expression of TMC1 started around P6 and rapidly increased to P12 (Fig. 5c). There was variability at P9 with good labelling in only one of three samples, which would suggest that there is a steep rise in expression and that some mice expressed more TMC1 than others at that stage. Tip labelling was acquired over a similar period; only one of three samples had distinct tip labelling at P9, where 16% of the short and intermediate tips were labelled, and at P12, 11% ± 0.02 (mean ± SEM, n = 3) of tips were labelled, whilst at P21 this was reduced to 8% ± 0.02 (mean ± SEM, n = 3). The number of particles per labelled tip was relatively stable from 1.6 per tip at P9 (in the one labelled sample), to 1.9 ± 0.14 (mean ± SEM, n = 3) per labelled tip at P12 and 1.4 ± 0.2 (mean ± SEM, n = 3) at P21. Labelling of the apical membrane at the base of the bundle was also detected (Figs 3–5). For comparison we include an unpublished image obtained during a previous study of LHFPL5 labelling^5^) showing that the LHFPL5 and TMC1 localise to the same region at the tip (Fig. 6) of P12.

## Discussion

The present study has shown, using custom made antibodies together with TEM at high resolution that (i) TMC1 protein is initially expressed between P3 and P6 in apical mouse hair bundles, rising to maximum expression between P9 and P12, and there is transitional localization to the kinocilium in the early stages; (ii) after the onset of expression, TMC1 is localized throughout developing hair bundles but becomes refined primarily to the tips of stereocilia between P6 and P12, where it remains at P21; (iii) TMC1 appears at the tip location before the onset of hearing but after the acquisition of LHFPL5 at the same location, and remains there post-weaning. The lack of labelling in the *Tmc1*^−/−^ by immunofluorescence and pre-embedding TEM also supports our conclusion that these data reflect the identification and localization of this protein using the custom made antibodies, and does not represent artefactual background labelling.

In TEM the localization of label to shorter stereociliary tips is consistent with TMC1 becoming part of the MET machinery at the lower end of the tip link during development. Thus, this study supports previous work that strongly suggests that TMC1 forms part of the MET channel, as suggested by Kawashima *et al*.^7^ and Pan *et al*.^8,9^, but is still consistent with the idea that it could be a chaperone for other components of the complex as previously suggested by Kim *et al*.^23^. The first detected expression in our data occurs between P3 and P6, as no labelling was detected at P3 but weak labelling at P6. The labelling then increases to reach its maximum at P12. In comparison with expression data from Kawashima *et al*.^7^, the onset of TMC1 labelling in apical regions in our material is similar (around P5-P6). However, compared with, Kurima *et al*.^19^ using mCherry-Tmc1 construct expression, our data suggest a later start for TMC1 appearance than theirs, where they observed punctate labelling, with some, but not all, at stereocilia tips by P3; however two factors may affect this: Firstly, there are changes in MET function and TMC1 expression along the cochleotopic axis^20,24^, so it is important to compare similar locations - the discrepancy may possibly reflect differences in the temporal maturation of hair cells in different regions and the location of data from Kurima *et al*.^19^ are not always specified so direct comparison is not possible. Their data also show labelling located in non-tip regions for P3 to P8, similar to our P9 data. Secondly, the sensitivity of the immunogold technique may be lower than fluorescence, so we may have missed the very earliest point of protein expression.

Our results can also be compared with the physiological data on the temporal and spatial maturation of the MET channel. The MET current elicited by bundle stimulation reaches maximal size at P4 in apical hair cells^24^ indicating TMC1 acquisition may have occurred by this stage. However, in *Tmc1*^−/−^ hair cells start to show reduced MET currents at P6, and the current declines to 0 by P10; this is potentially consistent with our data in that prior to P6 MET could be based primarily on TMC2 and a switchover occurs around P6. The loss of MET function at P10 in *Tmc1*^−/−^, suggesting that this age is where TMC1 exerts most effect in development, is consistent with the rise to maximal TMC1 expression between P9 and P12 in the wild type mice in our data. Thus our data are reasonably consistent with other studies in terms of time course of expression. The physiological data suggesting gradients in the quantity of TMC1 expression from apex to base^20^ would be amenable to further immunogold studies of this protein although that was beyond the scope of the present study.

A relationship between LHFPL5 and TMC1 is supported by the similarity of their localization. Both TMC1 from P9 and LHFPL5 from P0 are present in the stereocilia tips at least up to P21^5^ and neither is localized to the tallest stereocilia tips to any great extent. However, LHFPL5 is present at the tips of stereocilia at least by P0 and is distributed between P3 and P9 on the shafts of the stereocilia. TMC1 is also distributed on the shafts at P9. Quantitatively, TMC1 tip labelling (number of particles per labelled tip), once established at P9, remains at approximately the same level at P12 but drops a small degree by P21. The percentage of short and intermediate tips labelled also drops between P12 and P21, similar to the reduction seen with LHFPL5. It may therefore be the case that LHFPL5 is involved with capturing and/or maintaining TMC1 at the correct location, but not *vice versa* since LHFPL5 is localized there prior to acquisition of TMC1. This supports the observations made previously that TMC1 does not localize in the bundle in the absence of LHFPL5^22^.

Although gold labelling is not present on all tips, as discussed in our previous studies of LHFPL5, there is a likelihood that we either miss some of the tip labelling relatively easily in TEM sections, or that the protein’s antigenic sites are difficult to access in the protein rich top of the stereocilium actin core and the MET complex because of steric hindrance and epitope masking. This could substantially reduce the labelling efficiency, especially where secondary antibodies conjugated to 10 nm gold particles are involved, which are large compared with the fluorescent molecular tags used for immunofluorescent labelling (see^5^ also discussion in^25^). Even with immunofluorescence, Kurima *et al*.^19^ reported that not all tips were labelled in their study, suggesting a similar problem for smaller fluorescent secondary antibodies.

Labelling detected in other regions than the tip, especially at earlier ages, presumably reflects intermediate stages in acquisition of TMC1. At P3 and P6, TMC1 appears to occur on the kinocilium. This may represent initial untargeted expression in the bundle, but refinement is completed by P12 as discussed above. TMC1 labelling is also found along the apical membrane below the stereocilia at all ages. This latter may represent a pool of TMC1 molecules available for turnover and re-acquisition in the stereocilia throughout the lifetime. This possibility would provide partial explanation of the recovery of fluorescent gentamicin uptake (gentamicin is believed to enter through functional MET channels) following noise damage that destroys tip links and causes temporary threshold shift^26^. In this scenario, tip links would also have to repair as suggested by *in vitro* studies^27,28^ and re-associate with MET complex proteins such as the TMC1.

Labelling was also found in supporting cells, although it was more variable than stereociliary labelling. Expression of TMC1 in supporting cells has been previously reported in one other study^29^, although these authors did not report stereocilia labelling. Most studies have focused primarily on hair cells, so it remains to be determined whether this labelling reflects real supporting cell expression or is an artefact; we did not find labeling of supporting cells in the knockout under the same conditions suggesting it could represent the protein, whilst variability in supporting cell labelling argues the converse.

In conclusion, our data provide strong ultrastructural evidence that TMC1 is located in close proximity to the lower end of the tip link and to the putative accessory protein LHFPL5, providing further evidence that supports its role as the MET channel itself or as a crucial component of the channel gating complex. The association with LHFPL5 spatially but not temporally further supports the concept that the latter is involved in acquisition/maintenance of TMC1 at the tips, but not *vice versa* since LHFPL5 is expressed at the tips at least 9 days earlier than TMC1.

## Materials and Methods

### Antibodies

The primary antibody was an affinity-purified rabbit polyclonal antibody to the N-terminal region of mouse TMC1 (aa 53–72, CDEETRKAREKERRRRLRRGA, as used by Kurima *et al*.^19^ custom made by Life Technologies –ThermoFisher Scientific, Lancashire, UK). The secondary antibodies were Alexa Fluor 568 goat anti-rabbit IgG (H + L) (Invitrogen Molecular Probes, Oregon, USA), and goat anti-rabbit IgG conjugated to 10 nm gold particles (British BioCell, Cardiff, UK). Actin in the stereocilia was detected using phalloidin-fluorescein isothiocyanate labelling (cat #P5282; Sigma-Aldrich, Dorset, UK) in some samples.

### Animals

CD/1 mice ranging from P3-P21 in age were bred and maintained in Keele University’s Central Animal facility and treated in accordance with the UK Animals (Scientific Procedures) Act of 1986. The *Tmc*1 knockout (*Tmc*1^−/−^) mice at P9 to P12 (kindly provided by R Fettiplace and M Beurg) were bred and maintained in the animal facility in the University of Wisconsin-Madison according to protocols approved by Institutional Animal Care and Use Committee of the University of Wisconsin-Madison.

### Fixation

For fixation and wild type studies, CD/1 mice were anaesthetised with an overdose of sodium pentobarbitone (IP; Pentoject, Animalcare Ltd, York), decapitated and fixed with 4% paraformaldehyde (PFA) in 0.1 M phosphate buffer (PB), pH 7.4. For P3 mice, the fixative was perfused through the internal acoustic meatus and for mice older than P3, a small hole was made in the apex and fixative was perfused through the round window, followed by immersion of the cochlea in the same fixative for 20–60 min at room temperature. For *Tmc1*^−/−^, animals were killed by decapitation and fixed by perfusion as above with 4% PFA in 0.1 M PB, pH 7.4 for 20 min. All the fixed cochleae were stored in 1/10^th^ concentration of the fixative at 4 °C until immunolabelling was performed.

### Immunofluorescence labelling

The fixed cochleae were washed in PB, dissected into cochlear spirals, permeabilized with 0.5% Triton X-100 in 0.01 M phosphate-buffered saline (PBS) at pH 7.4 for 30 min, immersed in 10% goat serum (GS – Sigma, Dorset, UK) in PBS (GS-PBS) for 1 h to block non-specific labelling, and incubated for 16–48 h at 4 °C in anti-TMC1 antibody (AB0144) diluted 1:25–1:50 in PBS containing 1% GS and 0.2% Tween 20 (1% GS-PBST). After rinsing in 1% GS-PBST, they were incubated for 2 h at room temperature in Alexa Fluor 568 (ThermoFisher, Lancashire, UK) goat anti-rabbit IgG (H + L) diluted 1:50, with or without phalloidin-fluorescein isothiocyanate labelled diluted 1:500–1:750, in 1% GS-PBST and washed in PBS. The labelled cochlear segments were mounted in our custom made anti-fade solution (10 mM *p-*phenylenediamine in 2% polyvinyl alcohol and 33% glycerol) with coverslips and viewed under a 100 X oil-immersion objective using a BioRad MRC 1024 confocal laser microscope.

### Pre-embedding immunogold labelling

The fixed cochleae were washed in PBS, dissected into whole cochlear spirals by removing the external bone, permeabilized with 0.5% Triton X-100 in PBS for 30 min, immersed in 10% GS-PBS for 1 h at room temperature to block non-specific labelling and incubated for 16–48 h at 4 °C in anti-TMC1 antibody (AB0144) diluted 1:25–1:50 in 1% GS-PBST. After rinsing in 1% GS-PBST, they were incubated in goat anti-rabbit IgG conjugated to 10 nm gold particles (British Biocell, Cardiff, UK) diluted 1:10 in 1% GS-PBST for 2 h at room temperature and washed in PBS. They were then fixed in 2.5% glutaraldehyde (EM grade, Agar Scientific, UK) in 0.1 M sodium cacaodylate buffer (NaCaco), pH 7.4 for 2 h, washed in NaCaco, fixed in 1% osmium tetroxide in NaCaco for 1 h, dehydrated through a graded series of ethanol (70%, 80%, 90%, 100%, dry 100%) for 15 min in each concentration, infiltrated in a mixture of ethanol and Spurr resin (Agar Scientific, UK), followed by pure Spurr resin and polymerised at 60 °C overnight. Semithin (250 nm) radial sections were cut at a position of 180° from the apex (approximately 80% of the full length of the organ of Corti from the basal end) using a Leica Ultracut UCT ultramicrotome (Solms, Germany) and collected on 200-mesh thin-bar copper grids. The sections were then examined using a JEOL JEM 1230 transmission electron microscope operated at an accelerating voltage of 100 kV.

For semi-quantitative analysis, gold particle numbers were counted in approximately 50 images for each age analysed. Four types of analysis were performed: (1) number of particles per height above cuticular plate (in bins of 0.1 μm); total number of particles per image; (3) percentage of short and intermediate stereocilia tips labelled; and (4) number of particles per labelled tip.

## Data Availability

Data and materials (where stocks remain) will be made available on request to the corresponding author. Data have been analysed using non-parametric statistics. Comparison of two samples has been done using the unpaired Wilcoxon (Mann-Whitney) test. All images have been processed using Adobe Photoshop 7®. No changes have been made other than brightness and contrast to optimize image clarity.
